# Long noncoding RNA MALAT1 promotes hepatic steatosis and insulin resistance by increasing nuclear SREBP-1c protein stability

**DOI:** 10.1038/srep22640

**Published:** 2016-03-03

**Authors:** Caifeng Yan, Jinfeng Chen, Nuoqi Chen

**Affiliations:** 1 Department of Endocrinology, Clinical Medical College of Yangzhou University, Yangzhou, China; 2 Department of Endocrinology, Zhangzhou Affiliated Hospital of Fujian Medical University, No. 59, Shengli Road, Xiangcheng District, Zhangzhou, China; 3 Department of Endocrinology, Zhangzhou Affiliated Hospital of Fujian Medical University, Zhangzhou, China

## Abstract

Metastasis-associated lung adenocarcinoma transcript 1 (MALAT1) is implicated in liver cell proliferation. However, its role in hepatic steatosis and insulin resistance remain poorly understood. The aim of this study was to investigate the effects of MALAT1 on hepatic lipid accumulation and its potential targets. As expected, MALAT1 expression is increased in hepatocytes exposed to palmitate and livers of ob/ob mice. Knockdown of MALAT1 expression dramatically suppressed palmitate-induced lipid accumulation and the increase of nuclear SREBP-1c protein in HepG2 cells. In addition, RNA immunoprecipitation and RNA pull-down assay confirmed that MALAT1 interacted with SREBP-1c to stabilize nuclear SREBP-1c protein. Finally, injection of si-MALAT1 prevented hepatic lipid accumulation and insulin resistance in ob/ob mice. In conclusion, our observations suggest that MALAT1 promotes hepatic steatosis and insulin resistance by increasing nuclear SREBP-1c protein stability.

The incidence of type 2 diabetes mellitus (T2DM) has increased drastically in recent years, which poses a serious threat to public health worldwide^1^. It is clear that insulin resistance participates in the development of this disease^2^. Due to playing a vital role in lipid metabolism and glucose metabolism, the liver is one of the main peripheral organs of insulin resistance^3^,^4^. Accumulating evidence indicates that lipid accumulation in the liver is closely associated with hepatic insulin resistance and T2DM^5^. However, the pathogenic regulation of hepatic steatosis remains unclear at the molecular level.

Sterol regulatory element-binding protein (SREBP)-1c is expressed abundantly in hepatocytes and is primarily activated by insulin^6^. As a key transcription factor to regulate the expression of genes required for hepatic lipogenesis, SREBP-1c is implicated in the development of fatty liver and dyslipidemia^7^. In the liver of obese non-alcoholic fatty liver disease (NAFLD) patients, the expression of SREBP-1c and its target genes is significantly increased^8^. In addition, nuclear SREBP-1c was significantly accumulated in the livers of ob/ob mice, which was induced by activation of hepatic ER stress^9^. Further study showed that overexpression of active SREBP-1c led to developing steatosis *in vivo* and *in vitro*^10^,^11^. In contrast, knockdown of hepatic SREBP-1c in obese mice caused a significant decrease in hepatic lipogenesis^12^. These data suggest that the increase of SREBP-1c expression and activity contributes to hepatic lipid accumulation and insulin resistance^13^.

Long noncoding RNAs (LncRNAs), >200 nucleotides, are involved in a variety of biological processes such as cell proliferation, apoptosis and differentiation^14^. Metastasis associated in lung adenocarcinoma transcript 1 (MALAT1) is a well-conserved lncRNAs and is implicated in some diseases including cancer^15^. Recently, MALAT1 has received attention for its role in progression of diabetic complications, as it was shown that MALAT1 dysregulation participates in the pathogenesis of diabetes-related microvascular disease and diabetic retinopathy^16^,^17^. Additionally, MALAT1 stimulated production of inflammatory cytokine in the endothelial cells treated with high glucose^18^. Deletion of MALAT1 expression obviously inhibited liver cell proliferation, which indicated that MALAT1 contributes to liver insulin resistance^19^. However, the effect of MALAT1 on hepatic steatosis has not been investigated. The present study was designed to evaluate the role of MALAT1 in hepatic lipid metabolism and insulin resistance both *in vitro* and *in vivo*.

## Results

### MALAT1 expression is increased in hepatocytes exposed to palmitate and livers of ob/ob mice

**Figure 1:**
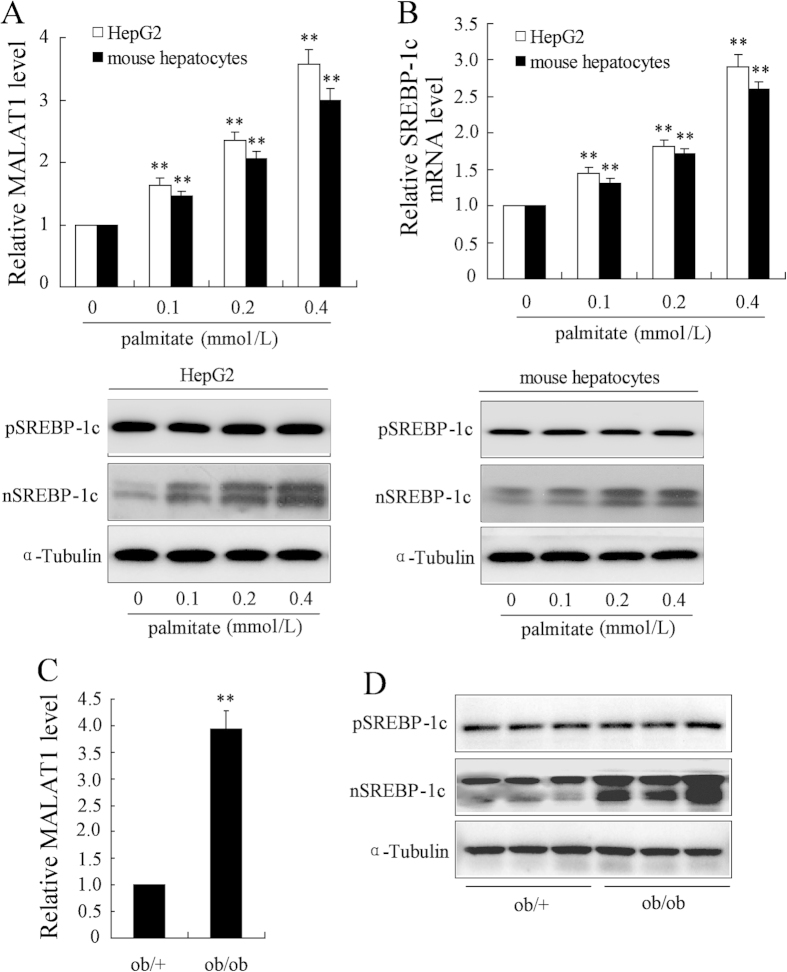
MALAT1 expression is increased in hepatocytes exposed to palmitate and livers of ob/ob mice. (**A**) HepG2 cells or mouse hepatocytes were treated with different concentration of palmitate (0, 0.1, 0.2, 0.4 mmol/L) for 24 h, then, MALAT1 levels (white bars, HepG2 cells; black bars, mouse hepatocytes) were measured. (**B**) HepG2 cells or mouse hepatocytes were treated with different concentration of palmitate (0, 0.1, 0.2, 0.4 mmol/L) for 24 h, then, SREBP-1c mRNA levels (white bars, HepG2 cells; black bars, mouse hepatocytes) and protein level (left, HepG2 cells; right, mouse hepatocytes) were determined. The MALAT1 levels (**C**) and SREBP-1c protein level (**D**) were measured in livers from ob/ob. **P < 0.01, compared to palmitate = 0  mmol/L or ob/ + mice.

Previous study showed that MALAT1 expression is significantly upregulated in endothelial cell of diabetic mice^18^. Here we investigated the MALAT1 expression pattern in HepG2 cells and primary mouse hepatocytes exposed with different doses of palmitate for 24 h. MALAT1 expression was dose-dependently increased in HepG2 cells and primary mouse hepatocytes (Fig. 1A). In addition to elevated MALAT1, palmitate treatment led to increased levels of the mRNA and nuclear SREBP-1c (Fig. 1B). We next evaluated these results *in vivo*. The MALAT1 levels were obviously elevated in livers from ob/ob mice (Fig. 1C). SREBP-1c expression was also increased in ob/ob mice (Fig. 1D), which was consistent with previous report^9^.

### Knockdown of MALAT1 reversed palmitate-induced lipid accumulation in HepG2 cells

**Figure 2:**
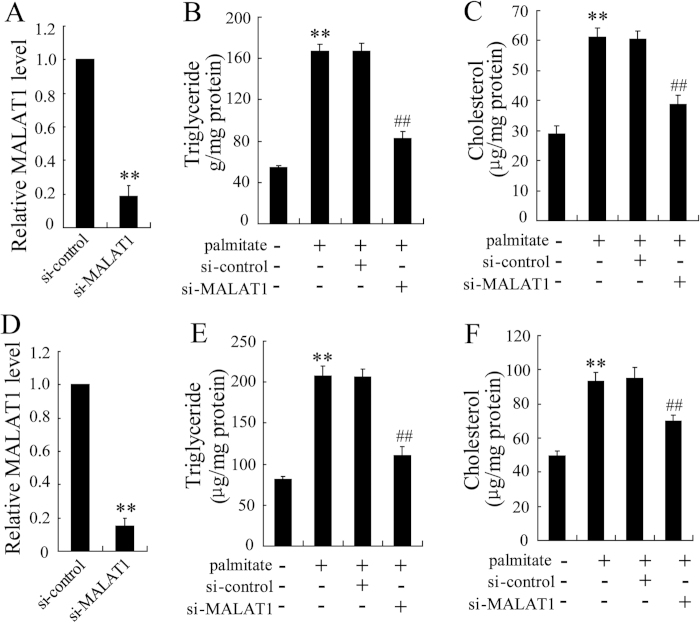
Knockdown of MALAT1 reversed palmitate-induced lipid accumulation in HepG2 cells. (**A**) HepG2 cells were transfected with si-control or si-MALAT1 for 48 h, then, MALAT1 expression was measured. After transfected with si-control or si-MALAT1 for 24 h, HepG2 cells were treated with palmitate (0.4 mmol/L) for 24 h. Then, intracellular levels of triglycerides (**B**) and cholesterol (**C**) were determined. (**D**) Mouse hepatocytes were transfected with si-control or si-MALAT1 for 48 h, then, MALAT1 expression was measured. After transfected with si-control or si-MALAT1 for 24 h, mouse hepatocytes were treated with palmitate (0.4 mmol/L) for 24 h. Then, intracellular levels of triglycerides (**E**) and cholesterol (**F**) were determined. **P < 0.01, compared to control. ^##^P < 0.01, compared to palmitate + si-control.

To establish the role of MALAT1 in lipid accumulation, we treated HepG2 cells and primary mouse hepatocytes with palmitate to mimic fatty acid overload conditions. Consistent with other’s report, palmitate induced high intracellular levels of triglycerides and cholesterol^12^. To identify whether MALAT1 was involved in the effect of palmitate on lipid accumulation, HepG2 cells were transfected with si-MALAT1. As shown in Fig. 2A, MALAT1 siRNA efficiently decreased the expression of MALAT1 in HepG2 cells. Notably, knockdown of MALAT1 significantly decreased the palmitate-induced lipid accumulation in HepG2 cells. Palmitate caused a 3-fold increase in intracellular levels of triglycerides, which were significantly reduced by silencing of MALAT1 (Fig. 2B). Similar results of intracellular levels of cholesterol were also observed (Fig. 2C). In addition, deletion of MALAT1 in primary mouse hepatocytes significantly decreased the palmitate-induced lipid accumulation (Fig. 2D–F).

### Knockdown of MALAT1 reversed palmitate-induced the increase of nuclear SREBP-1 protein in hepatocytes

**Figure 3:**
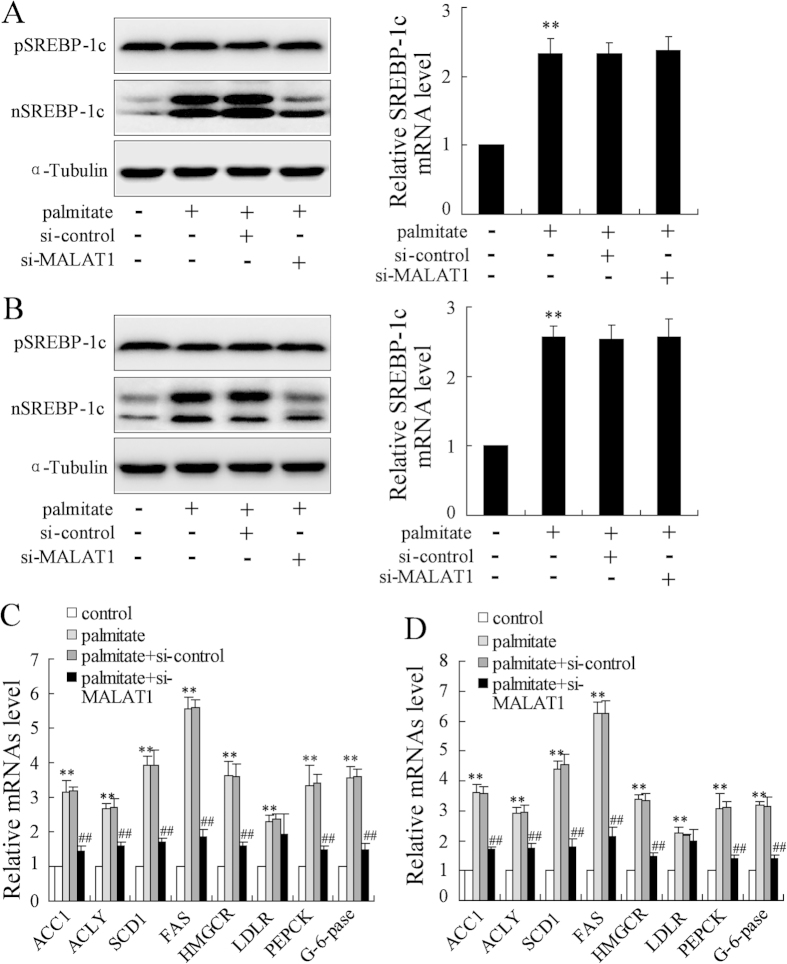
Knockdown of MALAT1 reversed palmitate-induced the increase of nuclear SREBP-1c protein in hepatocytes. After transfected with si-control or si-MALAT1 for 24 h, HepG2 cells were treated with palmitate (0.4 mmol/L) for 24 h. Then, the precursor form (p-), nuclear SREBP-1c (nSREBP-1c), and SREBP-1c mRNA expression (**A**) as well as ACC1, ACLY, SCD1, FAS, HMGCR, LDLR, PEPCK and G-6-pase mRNA expression (**C**) were measured. After transfected with si-control or si-MALAT1 for 24 h, primary mouse hepatocytes were treated with palmitate (0.4 mmol/L) for 24 h. Then, the precursor form SREBP-1c (pSREBP-1c), nuclear SREBP-1c (n-SREBP-1c) and SREBP-1c mRNA expression (**B**) as well as ACC1, ACLY, SCD1, FAS, HMGCR, LDLR, PEPCK and G-6-pase mRNA expression (**D**) were measured. **P < 0.01, compared to control. ^##^P < 0.01, compared to palmitate + si-control.

As shown in Fig. 3A, the reduction of MALAT1 expression abolished palmitate-enhanced nuclear SREBP1c protein level but had no effect on SREBP1c precursor and SREBP1c mRNA in HepG2 cells (Fig. 3A). Similar results were observed in primary mouse hepatocytes (Fig. 3B). Consistent with this, MALAT1 knockdown resulted in a significant decrease in the expression of SREBP-1c target genes, including ACC1, ACLY, SCD1 and FAS in HepG2 cells (Fig. 3C) and primary mouse hepatocytes (Fig. 3D) exposed to palmitate. These results indicated that knockdown of MALAT1 reversed palmitate-induced the increase of nuclear SREBP-1c expression and transcriptional activity in hepatocytes. Besides, MALAT1 knockdown decreased the expression of HMGCoA reductase (HMGCR) and gluconeogenic genes such as PEPCK and G-6-pase (Fig. 3C,D).

### MALAT1 upregulates SREBP-1c expression by stabilizing SREBP-1c protein in hepatocytes

**Figure 4:**
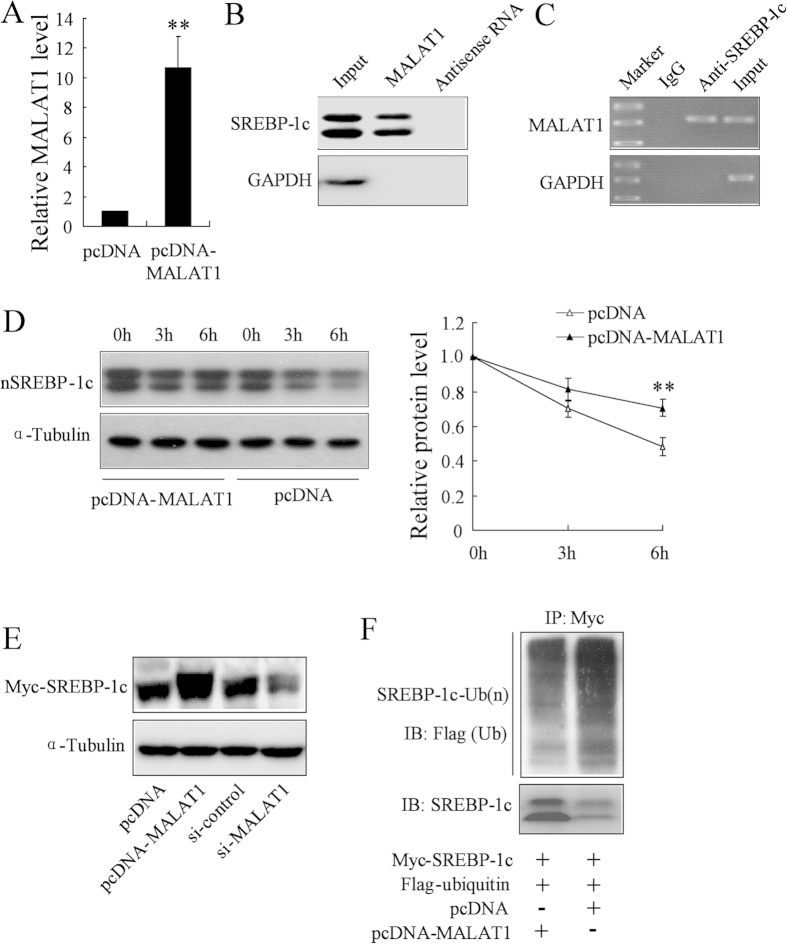
MALAT1 upregulates SREBP-1c expression by stabilizing SREBP-1c protein in hepatocytes. (**A**) HepG2 cells were transfected with pcDNA-MALAT1 and pcDNA for 48 h, and MALAT1 expression was measured. (**B**) MALAT1 or antisense RNA was incubated with nucleoprotein from HepG2 cell extracts, and the SREBP-1c protein was assayed by Western blot. A non-specific protein (GAPDH) was used as the control. (**C**) RIP experiments were performed in HepG2 cells using a SREBP-1c antibody or non-specific IgG, and specific primers were used to detect MALAT1 and GAPDH. (**D**) HepG2 cells were transfected with pcDNA-MALAT1 and pcDNA for 48 h, followed by exposure to cycloheximide (CHX 50 mg/ml) for 0, 3, or 6 h. The nuclear SREBP1c (nSREBP-1c) was measured by Western blot. (**E**) The Myc-tagged nuclear form of SREBP-1c (Myc-SREBP-1c) and pcDNA-MALAT1 or Myc-SREBP-1c and si-MALAT1 were transiently transfected in HEK293 cells for 48 h. The protein level of SREBP-1c was measured. (**F**) Flag-ubiquitin was coexpressed in HepG2 cells with myc-SREBP-1c and pcDNA-MALAT1 with treatment of MG132 (10 μM) for 6 h. Ubiquitinated -SREBP-1c protein was immunoprecipitated using Myc-Tag antibody and further detected with Anti-Flag antibody. **P < 0.01, compared to pcDNA.

Previous studies have confirmed that lncRNAs could directly interact with target proteins and increase their stability^20^. To investigate whether MALAT1 regulates SREBP-1c expression in such a manner, we overexpressed MALAT1 in HepG2 cells (Fig. 4A). Then, RIP and RNA pull-down assays were performed to identify whether SREBP-1c associated with MALAT1. As shown in Fig. 4B, MALAT1 was selectively interacting with nuclear SREBP-1c. SREBP-1c was measured by Western blot analysis in RNA pull-down assays. We also observed MALAT1 enrichment in RIP using SREBP-1c antibody in nuclear extracts from HepG2 cells (Fig. 4C).

We further explored whether MALAT1 increased SREBP-1c expression at the post-transcriptional level. We transfected pcDNA-MALAT1 in HepG2 cells for 24 h, then treated with the protein synthesis inhibitor cycloheximide (CHX) for 0, 3 or 6 h. Overexpression of MALAT1 inhibited degradation of nuclear SREBP-1c protein in HepG2 cells treated with CHX (Fig. 4D). To further analyze the effect of MALAT1 on nuclear SREBP-1c protein levels, the Myc-tagged nuclear form of SREBP-1c was overexpressed in HEK293 cells by transient transfection. As shown in Fig. 4E, overexpression of MALAT1 caused accumulation of the nuclear form of SREBP-1c, while depletion of MALAT1 decreased accumulation of the nuclear form of SREBP-1c. In addition, we found MALAT1 overexpression inhibited the ubiquitination of SREBP-1c (Fig. 4F). These results indicated that MALAT1 stabilized SREBP-1c by preventing ubiquitin-mediated degradation.

### SREBP-1c is required for MALAT1-induced intracellular lipid accumulation

**Figure 5:**
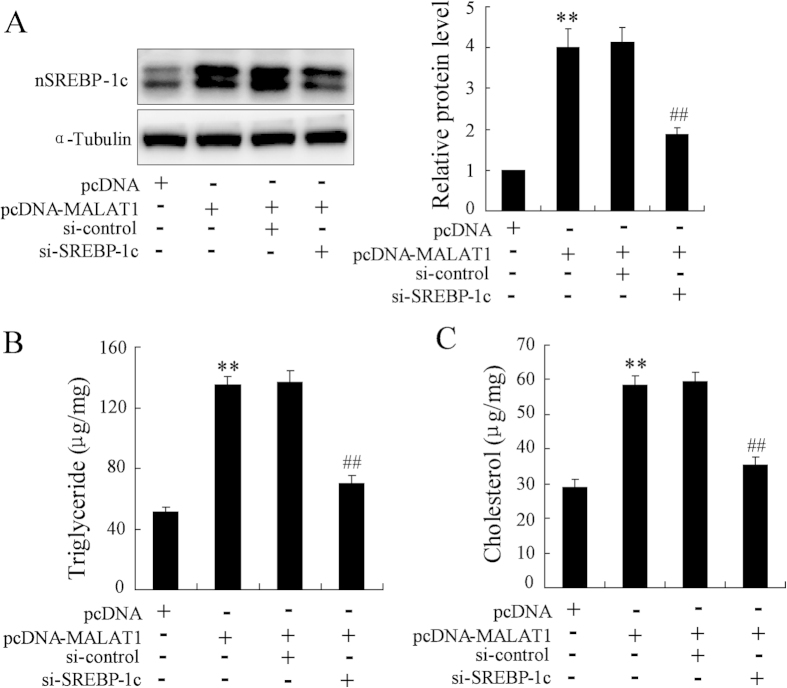
SREBP-1c is required for MALAT1-induced intracellular lipid accumulation. HepG2 cells were transfected pcDNA-MALAT1 and si-MALAT1 for 48 h, then nuclear (n-) SREBP-1c expression (**A**) intracellular levels of triglycerides (**B**) and cholesterol (**C**) were measured. **P < 0.01, compared to pcDNA. ^##^P < 0.01, compared to pcDNA-MALAT1 + si-control.

SREBP-1c is a key transcription factor that regulates the development of fatty liver and dyslipidemia^21^. We therefore studied whether SREBP-1c activation is required for MALAT1-induced lipid accumulation. Overexpression of MALAT1 in HepG2 cells increased nuclear SREBP1c protein level, which was reversed by transfection of SREBP-1c siRNA (Fig. 5A). In the cells transfected with si-control, overexpression of MALAT1 increased intracellular levels of triglycerides and cholesterol. However, lipid accumulation was weakened in HepG2 cells transfected with si-SREBP-1c (Fig. 5B,C). These results suggested that SREBP-1c mediates MALAT1-induced intracellular lipid accumulation.

### Knockdown of MALAT1 expression reversed aggregation lipid in ob/ob mouse liver

**Figure 6:**
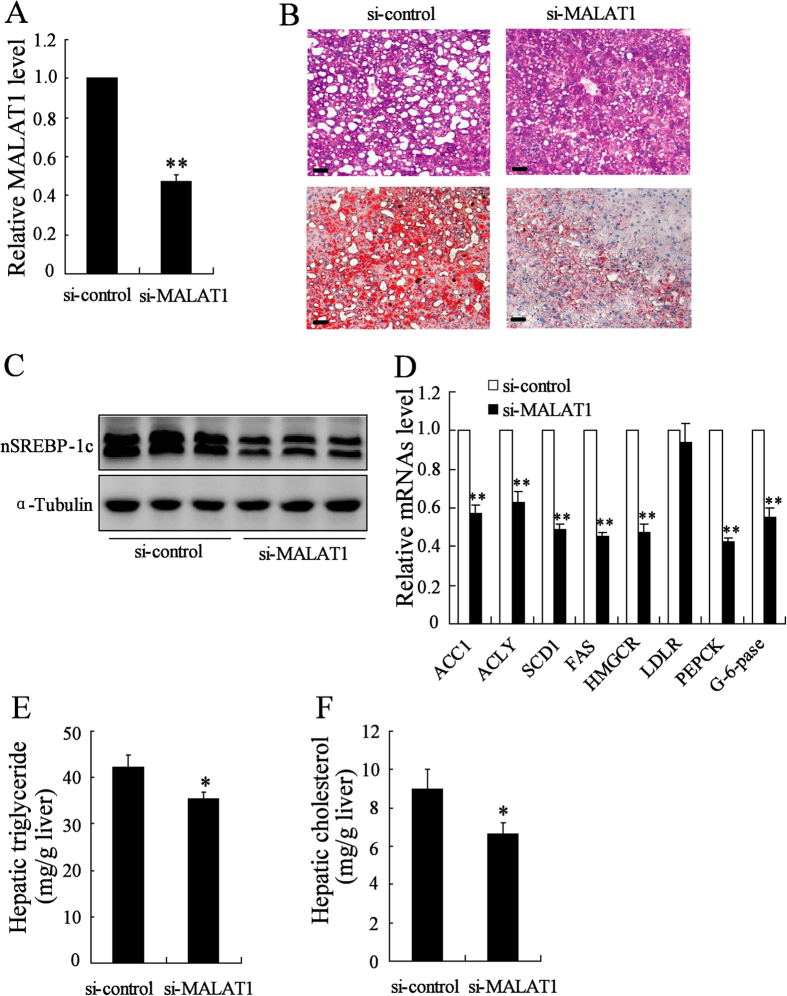
Knockdown of MALAT1 expression reversed aggregation lipid in male ob/ob mouse liver. (**A**) ob/ob mice were injected with si-control or si-MALAT1 daily for 10 days, then, MALAT1 expression in liver was determined (n = 5 per group). (**B**) ob/ob mice were injected with si-control or si-MALAT1 daily for 10 days, then, Liver sections were stained with H-E (top row) or Oil red O (bottom row). Original magnification, ×20. Scale bar = 50 μm (n = 3 per group). (**C**) The nuclear SREBP-1c (nSREBP-1c) expression was measured in liver from ob/ob mice injected with si-control or si-MALAT1 daily for 10 days. (**D**) ACC1, ACLY, SCD1, FAS, HMGCR, LDLR, PEPCK and G-6-pase mRNA expression was measured in liver from ob/ob mice injected with si-control or si-MALAT1 daily for 10 days (n = 3 per group). Hepatic triglycerides (**E**) and cholesterol content (**F**) were measured in liver from ob/ob mice injected with si-control or si-MALAT1 daily for 10 days (n = 6 per group). (E) **P < 0.01, compared to si-control.

To explore the role of LncRNA MALAT1-induced lipid accumulation in the liver, we treated ob/ob mice with si-MALAT1 daily for 10 days. MALAT1 expression in the si-MALAT1 injection group was significantly reduced in liver (Fig. 6A). The ratio of liver to body weight was significantly lower in si-MALAT1-injected mice (4.8 ± 0.32%) than that of si-control-injected mice (6.7 ± 0.43%). Meanwhile, a significant decrease in lipid accumulation in the liver in si-MALAT1-injected mice compared to si-control-injected mice (Fig. 6B).

We next determined whether si-MALAT1 effectively reduced nuclear SREBP-1c protein level *in vivo* in the ob/ob mouse liver. As shown in Fig. 6C, the amount of nuclear SREBP-1c was reduced in the livers of si-MALAT1-injected ob/ob mice compared with si-control-injected ob/ob mice. Meanwhile, the levels of ACC1, ACLY, SCD1, FAS, HMGCR, PEPCK and G-6-pase were strongly reduced in the livers of si-MALAT1-treated ob/ob mice (Fig. 6D).

### MALAT1 knockdown improves insulin sensitivity in ob/ob mice

**Figure 7:**
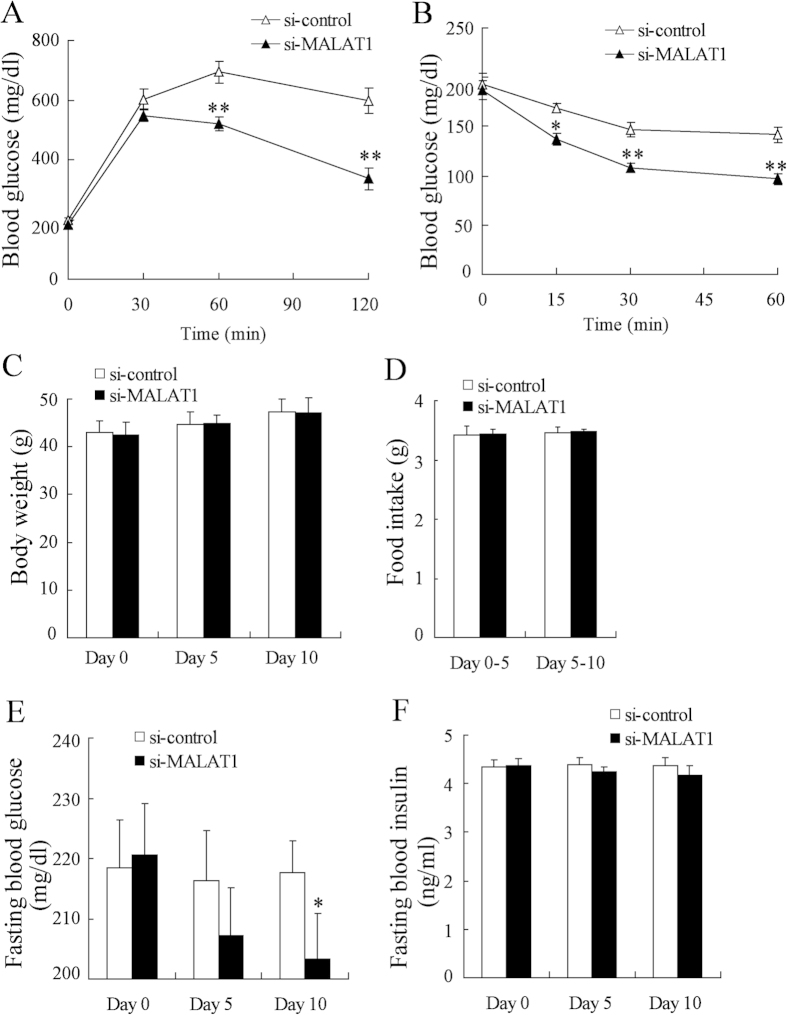
MALAT1 knockdown improves insulin sensitivity in male ob/ob mice. ob/ob mice were injected with si-MALAT1 (n = 8) or si-control (n = 8) daily for 10 days, then, IPGTT (**A**) and ITT (**B**) was measured. Body weight (**C**), food intake (**D**), fasting blood glucose levels (**E**) and fasting blood insulin levels (**F**) were measured after fasting the animals for 4 h on Day 0, Day 5 and Day 10 (n = 8 per group). *P < 0.05, **P < 0.01, compared to si-control.

Because hepatic steatosis has often been associated with hepatic insulin resistance^22^, we measured the effect of MALAT1 knockdown on insulin sensitivity. Figure 7A showed the effect of si-MALAT1 on IPGTT in ob/ob mice. Blood glucose levels of si-MALAT1-injected ob/ob mice were lower after intravenous glucose loading compared with si-control-injected mice. After administration of insulin (2 U/kg), si-MALAT1-injected ob/ob mice also exhibited lower levels of blood glucose than si-control-injected mice (Fig. 7B). Downregulation of MALAT1 expression in the liver had no effect on body weight and food intake of ob/ob mice (Fig. 7C,D). However, MALAT1 knockdown significantly decreased fasting blood glucose after 10 days injection (Fig. 7E). MALAT1 knockdown did not affect fasting blood insulin levels (Fig. 7F). These results indicated that improvement of glucose intolerance in si-MALAT1-injected ob/ob mice was due to enhancing insulin sensitivity but not stimulating insulin secretion.

## Discussion

The present study firstly demonstrated that excess palmitate increased LncRNA MALAT1 expression, activated SREBP-1c, and induced intracellular lipid accumulation in hepatocytes. We found that MALAT1 expression is increased in hepatocytes exposed to palmitate and livers of ob/ob mice. We also found that inhibition of the MALAT1 expression decreased nuclear SREBP1c level and lipid accumulation both *in vitro* and *in vivo*. In addition, the reduction of MALAT1 in the liver improved insulin sensitivity in ob/ob mice.

LncRNA MALAT1 has been discovered to be involved in liver cell proliferation^19^. However, the function of MALAT1 in the improvement of hepatic steatosis has not been studied. Our data demonstrated that the expression of MALAT1 was obviously increased in hepatocytes under fatty acid overload conditions. In addition, MALAT1 expression is markedly elevated in the livers of ob/ob mice. Gene silencing of MALAT1 attenuated lipid accumulation in palmitate-treated HepG2 cells. In ob/ob mice, knockdown of MALAT1 significantly reduced liver lipids and promoted insulin sensitivity. More importantly, overexpression of MALAT1 leading to lipid accumulation. These findings establish a novel role of MALAT1 in hepatic steatosis and insulin resistance.

The data of the present study additionally demonstrated that increased MALAT1 is associated with nuclear SREBP-1c protein level. Deletion of MALAT1 abolished the increase of nuclear SREBP-1c protein level induced by palmitate but had no effect on SREBP-1c precursor and SREBP-1c mRNA. Therefore, MALAT1 regulated SREBP-1c expression at post-transcriptional level. Accumulating evidence has demonstrated that lncRNAs may interact with proteins and change the activity of these proteins^20^,^23^. MALAT1 is localized in the nuclear, implicating that its function serves as protein-coding RNAs^24^. Our data showed that MALAT1 directly bound nuclear SREBP-1c protein and increased stabilization of SREBP-1c protein. Our results also demonstrated that knockdown of MALAT1 abolished the increase of expression of ACC1, ACLY, SCD1 and FAS, which were the target genes of SREBP-1c^25^. Furthermore, overexpression of MALAT1 caused accumulation of the nuclear form of SREBP-1c, whereas depletion of MALAT1 decreased accumulation. Interestingly, MALAT1 overexpression inhibited the ubiquitination of SREBP-1c. These results suggested that MALAT1 knockdown had effect on the protein instability of nSREBP-1c via the ubiquitin proteasome pathway. However, whether other proteins are involved in interaction of MALAT1 and SREBP-1c requires further study.

SREBP-1c is the critical transcription factor that regulates cholesterol and fatty acid synthesis^7^. In obese patients, increased SREBP-1c expression was observed in liver^26^. Inhibition of SREBP-1c could suppress hepatic lipogenesis and lipid accumulation in liver^27^. Our present study found that downregulation of SREBP-1c expression effectively abolished the increase of intracellular levels of triglycerides and cholesterol induced by MALAT1. These finding indicated that the effect of MALAT1 on intracellular lipid accumulation is dependent on SREBP-1c. Moreover, we also found that MALAT1 had the effect on SREBP-2 mRNA stability (data not shown). Future studies are necessary to clarify how MALAT1 regulated SREBP-2 expression *in vitro* and *in vivo*.

In summary, MALAT1 induced hepatic lipid accumulation and insulin resistance by increasing SREBP-1c and target genes expression. This study suggested inhibition of MALAT1 has potential for the treatment of obesity and type 2 diabetes.

## Materials and Methods

### Reagents

DMEM, Trizol and Lipofectamine 2000 transfection reagent were purchased from Invitrogen Life Technologies (Grand Island, NY, USA). Fetal bovine serum (FBS) was obtained from GIBCO (Burlington, ON, USA). Palmitate, pentobarbital sodium salt, Cycloheximide (CHX), *α*-tubulin antibody (T8203) Anti-myc Tag antibody (SAB4301135) and Anti-Flag Tag antibody (SAB1305535) were purchased from Sigma (Saint Louis, USA). Antibody against SREBP-1c (sc-13551) was obtained from Santa Cruz Biotechnology (Santa Cruz, CA, USA). The Flag-ubiquitin and myc-SREBP1c expression vectors were a kind gift from Dongming Su (Nanjing Medical University, Nanjing, China).

### Cell culture

HepG2 cells were purchased from ATCC and maintained in DMEM supplemented with 10% FBS. Cells were cultured at 37 °C in a humidified atmosphere containing 95% air and 5% CO_2_. To generate the *in vitro* model of high fat-induced insulin resistance, HepG2 cells were treated with palmitate for 24 h as previously described^28^.

### Primary isolation and culture of hepatocytes

Hepatocytes were isolated and cultured from the livers of male C57BL/BKS mice as described previously^29^. Cell viability of primary hepatocytes was evaluated by the trypan blue exclusion test and was always higher than 70%.

### Animal studies

Mice were housed under 12/12 hr light/dark cycles and free access to water and a standard chow diet containing 60% carbohydrate, 13% fat, and 27% protein on a caloric basis. All animals studied were 8- to 12-week-old male ob/+ or ob/ob mice obtained from Model Animal Research Center of Nanjing University, China. Mice were anesthetized with pentobarbital sodium salt (35 mg/kg) and injected through the tail vein with 2.5 mg/kg body weight of lipid nanoparticles-formulated si-control or si-MALAT1 as described previously^30^. Body weight, fasting blood glucose levels and fasting blood insulin levels were measured after fasting the animals for 4 h (starting from 9:00 AM) on Day 0 (before injection), Day 5 and Day 10. Blood glucose concentrations were measured with the Hexokinase Method (Thermo Fisher Scientific, Lafayette, CO, USA). Blood insulin was measured using a rat/mouse insulin ELISA kit (Millipore, Billerica, MA). All animal studies were performed according to guidelines established by the Research Animal Care Committee of Yangzhou University, China. Animals were treated humanely, using approved procedures in accordance with the guidelines of the Institutional Animal Care and Use Committee at Yangzhou University, China.

### Measurement of intracellular cholesterol and triglyceride

Lipids in cultured HepG2 cells and primary mouse hepatocytes were extracted using a Folch extraction of lipids and resuspension in 0.1% Triton X-100 as described previously^28^. The intracellular cholesterol and triglyceride in extracted lipids were measured using the reagents from Cayman Chemical (Ann Arbor, MI) according to the manufacturer’s instruction. The hepatic triglyceride and cholesterol content were normalized with tissue weight.

### Small interfering RNA

Small interfering RNA specific for MALAT1 (si-MALAT1), SREBP-1c (si-SREBP-1c) and control siRNA (si-control) was synthesized (Ribobio, Guangzhou, China) and transfected using Lipofectamine 2000 in HepG2 cells and mouse hepatocytes. The sequences of si-MALAT1 were: 5′-CUUAUCAAUUCACCAAGGATTdTd T-3′and 3′-dTdTUCCUUGGUGAAUUGAUAAGTA-5′(for human); 5′-GGCUAAACAUC UAGGGUAATTdTdT-3′and 3′-dTdTUUAC CCUAGAUGUUUAGCCTT-5′(for mouse). The sequences of si-SREBP-1c were: 5′-GGAGGCUUCUCUACAGGAATTdTdT-3′and 3′-dTdT UUCCUGUAGAGAAGCCUCCTT-5′(for human); 5′-GAUAUCUGCAGUUGCUAAAdT dT-3′and 3′-dTdTUUUAGCAACUGCAGAUAUC-5′(for mouse).

### Real-time PCR assay

The mRNA was quantified by real-time PCR using a LightCycler480 II Sequence Detection System (Roche, Basel, Switzerland). All data were analyzed using β-Actin gene expression and GAPDH as internal standard. The sequences of primers used are available in supplemental table 1.

### Western blot analysis

HepG2 cells, primary mouse hepatocytes and ob/ob liver tissue were lysed with ice-cold lysis buffer containing: 50 mmol/l Tris–HCl, pH 7.4; 1% NP-40; 150 mmol/l NaCl; 1 mmol/l EDTA; 1 mmol/l phenylmethylsulfonyl fluoride; and complete proteinase inhibitor mixture (one tablet per 10 ml; Roche Molecular Biochemicals, Indianapolis, IN, USA). Individual immunoblots were probed with a rabbit anti-SREBP-1c antibody diluted 1:1000; and mouse anti-*α*-tubulin antibody diluted 1:5000.

### RNA immunoprecipitation (RIP)

RIP experiments were performed using the Magna RIP^TM^ RNA-binding protein immunoprecipitation kit (Millipore, Bedford, MA, USA) according to the manufacturer’s instructions. The antibody for RIP assays of SREBP-1c was diluted 1:50. Co-precipitated RNAs were detected by quantitative RT-PCR.

### RNA pull-down assay

MALAT1 was *in vitro* transcribed from vector pSPT19-MALAT1 and biotin-labeled with the Biotin RNA Labeling Mix (Roche Diagnostics, Indianapolis, IN) and SP6 RNA polymerase (Roche), and purified with an RNeasy Mini Kit (Qiagen, Valencia, CA). One milligram of nucleoprotein from HepG2 cell extracts was then mixed with 50 pmol of biotinylated RNA, incubated with streptavidin agarose beads (Invitrogen, Carlsbad, CA), and washed. The retrieved proteins were detected using standard Western blot analysis.

### Ubiquitination Assay

HepG2 cells were transfected with plasmids encoding Myc-SREBP1c, Flag-ubiquitin, and pcDNA-MALAT1. After transfection for 48 h, the cells were incubated with MG132 (10 μM) for 6 hours and lysed with cold RIPA buffer. Equal amounts of total cell lysates were incubated with the Myc antibodies for 2 hours at 4 °C. Immunocomplexes were collected using protein-A sepharose beads for 1 hour at 4 °C. Further, the immunoprecipitates were washed with RIPA buffer and subjected to SDS-polyacrylamide gel electrophoresis (SDS-PAGE) followed by western blotting analyses with anti-Flag antibodies.

### Expression plasmids construction

The MALAT1 expression plasmid (pcDNA-MALAT1, nucleotides 3207–8411 bp) was constructed as previous report^31^. The sequences of primers were as follow: MALAT1, 5′-TGAGTCGAGCTCTGCCAAGTCCTGGAGAAATAGTAG-3′(forward) and 5′-AGTCATGGGCCCTGAAGACAGATTAGTAAAAGCA-3′(reverse). he plasmid of pcDNA-MALAT1 was sequenced and confirmed to be correct.

### Histological analysis

For H-E, liver tissues were fixed in 10% neutral-buffered formalin, embedded in paraffin, and cut into 4 μm sections. For Oil red O staining, liver was frozen in liquid nitrogen and cut into 8 μm sections. Section were stained and analyzed at ×20 magnification using a Leica DMRB microscope.

### Intraperitoneal glucose tolerance test (IPGTT) and insulin tolerance test (ITT)

IPGTT was performed by an intraperitoneal injection of D-glucose (2 g/kg body weight, i.p.) after 6 h fast. Blood glucose levels were measured at 0, 30, 60, 90, and 120 minutes post injection. For ITT, mice were fasted for 6 h and then injected with human insulin (Novo-Nordisk, Bagsværd, Denmark) at 0.75 U/kg body weight. Blood glucose levels were measured at 0, 15, 30, and 60 minutes.

### Statistical analysis

Statistical analyses were performed using statistical analysis software SPSS 13.0. Data were expressed as the mean ± SD. Analysis of variance (ANOVA) was used to determine the statistical differences among the groups. A P value of less than 0.05 and are provided in the figures. A *P* value < 0.05 was considered statistically significant.

## Additional Information

**How to cite this article**: Yan, C. *et al.* Long noncoding RNA MALAT1 promotes hepatic steatosis and insulin resistance by increasing nuclear SREBP-1c protein stability. *Sci. Rep.* **6**, 22640; doi: 10.1038/srep22640 (2016).

## Supplementary Material

Supplementary Information
