# Supplementary material for: Long noncoding RNA MALAT1 promotes hepatic steatosis and insulin resistance by increasing nuclear SREBP-1c protein stability
Source: Sci Rep. 2016 Mar 3;6:22640. doi: 10.1038/srep22640 (PMC4776244; doi:10.1038/srep22640)
Supplement: Supplementary Information [file srep22640-s1.pdf]

Long noncoding RNA MALAT1 promotes hepatic steatosis and insulin resistance by increasing nuclear SREBP-1c protein stability

Caifeng Yan[1], Jinfeng Chen[2], Nuoqi Chen[3*]

1Department of Endocrinology, Clinical Medical College of Yangzhou University, Yangzhou, China; [2]Department of Endocrinology, Zhangzhou Affiliated Hospital of Fujian Medical University, No. 59, Shengli Road, Xiangcheng District, Zhangzhou, China; [3]Department of Endocrinology, Zhangzhou Affiliated Hospital of Fujian Medical University, Zhangzhou, China

*Co-corresponding author: Nuoqi Chen, Department of Endocrinology, Zhangzhou Affiliated Hospital of Fujian Medical University, No. 59, Shengli Road, Xiangcheng District, Zhangzhou, 363000, China.

Tel/ Fax: 86-596-2082030

E-mail: chennuoqi1016@sina.com

Table 1 Sequences of primers for real-time PCR.

Primers for real-time PCR		Sequences
MALAT1 (human)	Sense	5'-CTTAAGCGCAGCGCCATTTT-3'
	Antisense	5'-CCTC CAAACCCCAAGACCAA-3'
MALAT1 (mouse)	Sense	5’-GAGTTCTAATTCTTTTTACTGCTCAATC-3’
	Antisense	5’-AGAGCAGAGCAGCGTAGAGC-3’
SREBP-1c (human)	Sense	5’-GGATTGCACTTTCGAAGACATG-3’
	Antisense	5’-ACTCTGG ACCTGGGTGTGCAAG-3’
SREBP-1c (mouse)	Sense	5’-GTGAGCCTGACAAGCAATCA-3’
	Antisense	5’-GGTGCCTACAGAGCAAGAG-3’
ACC1 (human)	Sense	5’-GAATGTTTGGGGATATTTCAG-3’
	Antisense	5’-TTCTGCTATCAGTCTGTCCAG-3’
ACC1 (mouse)	Sense	5’-GCTTATTGATCAGTTATGTGGCC-3’
	Antisense	5’-CTGCAGGTTCTCAATGCAAA-3’
ACLY (human)	Sense	5’-GCCCATCCCCAACCAGCCAC-3’
	Antisense	5’-TTGCAGGCGCCACCTCATCG-3’
ACLY (mouse)	Sense	5’-AAGAAGGAGGGGAAGCTGAT-3’
	Antisense	5’TCGCATGTCTGGGTTGTTTA3’
SCD1 (human)	Sense	5’-CCTCTACTTGGAAGACGACATTCG-3’
	Antisense	5’-GCAGCCGAGCTTTGTAAGAGC-3’
SCD1 (mouse)	Sense	5’-AAGATATTCACGACCCCACC-3’
	Antisense	5’-CAGCCGTGCCTTGTAAGTTC-3’
FAS (human)	Sense	5’-CAAGAACTGCACGGAGGTGT-3’
	Antisense	5’-AGCTGCCAGAGTCGGAGAAC-3’
FAS (mouse)	Sense	5’-GATCCTGGAACGAGAACAC-3’
	Antisense	5’-AGACTGTGGAACACGGTGGA-3’
HMGCR (human)	Sense	5’-ACATTGTCACCGCCATCTACATTGC-3’
	Antisense	5′-GGCTTGCTGAGGTAGTAGGTTGGT-3′
HMGCR (mouse)	Sense	5′-TACAACGCCCACGCAGCAAACA-3’

	Antisense	5′-ACCAACCTTCCTACCTCAGCAA-3′
LDLR (human)	Sense	5′-CTGTCTCTGTTGCGGATACCAAGG-3′
	Antisense	5′-GCGAGTAGATGTCCACACCATTCA-3′
LDLR (mouse)	Sense	5′-AGCCATTTTCAGTGCCAATC-3′
	Antisense	5′-GAGGAGGGCTGTTGTCTCAC-3′
PEPCK (human)	Sense	5’-TGAGCTGTGTCAGCCTGATCAC-3’
	Antisense	5’-ACCGTCTTGCTTTCGACCTG-3’
PEPCK (mouse)	Sense	5’-GAGAAAGCATTCAACGCCAGG-3’
	Antisense	5’-CACAGATATGCCCATCCGAGTC-3’
G-6-pase (human)	Sense	5’-GAGATCATCTCCTTCGGAAGCG-3’
	Antisense	5’-TTAGTTATGCCCAGGATCAGCATG-3’
G-6-pase (mouse)	Sense	5’-AGCTCCGTGCCTATAATAAAGCAG-3’
	Antisense	5’-CATACGTTGGCTTTTTCTTTCCTC-3’
GAPDH (human)	Sense	5’-ATGACATCAAGAAGGTGGTG-3’
	Antisense	5’-CATACCAGGAAATGAGCTTG-3’
GAPDH (mouse)	Sense	5’-GGCATTGTGGAAGGGCTCAT-3’
	Antisense	5’-GACACATTGGGGGTAGGAACAC-3’
β-Actin (human)	Sense	5'- GCAAGTGCTTCTAGGCGGAC-3'
	Antisense	5'- AAGAAAGGGTGTAAAACGCAGC-3'
β-Actin (mouse)	Sense	5'-AGGCCAACCGTGAAAAGATG-3'
	Antisense	5'-AGAGCATAGCCCTCGTAGATGG-3'
